# Modeling Sediment Transport Potential and Connectivity to Support Sustainable Hydropower Development in Central Asia

**DOI:** 10.1007/s00267-026-02598-8

**Published:** 2026-09-15

**Authors:** Hannah Schwedhelm, Jan De Keyser, Tobias Siegfried, Sandra Krone, Antonia Dallmeier, Roser Casas-Mulet, Nils Rüther

**Affiliations:** 1 Technical University of Munich, Chair of Hydraulic Engineering, TUM School of Engineering and Design, Munich, Germany; 2 BOKU University, Institute of Hydraulic Engineering and River Research, Department of Landscape, Water and Infrastructure, Vienna, Austria; 3 hydrosolutions GmbH, Zurich, Switzerland

**Keywords:** hydromorphology, Aral Sea basin, CASCADE, hydropower sustainability, sediment management

## Abstract

Hydropower development exerts strong pressure on river systems by fragmenting river basins, trapping sediments and creating downstream sediment starvation. These alterations severely degrade river ecosystems through losses in habitat abundance and deterioration of substrate composition. As hydropower continues to expand, there is an urgent need for informed sediment management to be included in hydropower planning. In Central Asia, where a high potential for hydropower development remains, hydromorphological data are limited, constraining the assessment of morphological processes. To address this data scarcity, this study creates for the first time a high-resolution dataset containing qualitative information on sediment transport and sediment connectivity in Central Asia. We modified a sediment connectivity model to work with openly available geospatial datasets and statistically variable input data. The simulation outputs were aggregated into spatially explicit probabilities of sediment transport, entrainment and deposition for five grain-size classes, along with connectivity indices. Comparisons with results from 2D numerical hydraulic modeling at four sites indicated an overall good agreement, although a consistent tendency toward overestimation. The resulting dataset facilitates the decision-making process in hydropower planning by informing three application domains: comparing hydropower sites, deciding on design and operational strategies, and evaluating ecological and morphological impacts. Although site-specific assessments and field data are crucial for individual hydropower projects, our dataset provides essential decision support for basin-wide hydropower development in Central Asia.

## Introduction

Hydropower development typically requires river damming or water abstraction, two aspects that are among the main stressors on rivers worldwide (Kondolf 1997; Nilsson et al. 2005; Winemiller et al. 2016; Best 2019). From a morphological perspective, the effects of large dams are widely understood and include ecosystem fragmentation, sediment trapping and sediment starvation downstream (Kondolf 1997; Nilsson et al. 2005; Schmutz and Moog 2018; Best 2019). For smaller dams and small hydropower plants, the effects are more variable and more difficult to quantify (Brandt 2000; Csiki and Rhoads 2010; Fencl et al. 2015; Pearson and Pizzuto 2015; Casserly et al. 2020; Casserly et al. 2021; Magilligan et al. 2021; Campos and Pedrollo 2023), but can comprise drastic ecological and morphological alterations (Gabbud and Lane 2016; Fantin-Cruz et al. 2020). Increasing sediment loads due to climate change (Li et al. 2021) further threaten hydropower infrastructure through reservoir infill and turbine degradation, affecting long-term sustainability (Li et al. 2022b). These challenges underscore the urgent need for improved process understanding and comprehensive data collection to support effective, multidisciplinary sediment management at both local and basin scales when designing and operating small and large-scale hydropower plants (Kondolf 1997; Nilsson et al. 2005; Fencl et al. 2015; Gabbud and Lane 2016; Winemiller et al. 2016).

Central Asia still holds considerable hydropower potential (Azimov and Avezova 2022; De Keyser et al. 2026b). The region’s rivers are already extensively utilized through numerous dams, reservoirs, and water diversion structures (Karthe et al. 2015; Zonn et al. 2020), yet many basins also contain pristine, highly biodiverse river stretches (Betz et al. 2016; CEPF 2017). Hence, balancing hydropower expansion with ecosystem integrity is a major regional challenge requiring regional-scale frameworks (De Keyser et al. 2026a). Sediment management is a critical component of sustainable hydropower development. Such management options must estimate the effects of sediment transport on proposed riverine structures, assess the morphological impacts upstream and downstream, and guide site selection for future water uses. However, the scarcity of sediment and morphological data in Central Asia severely hinders this management process. De Keyser et al. (2023) addressed the overall data scarcity in Central Asia by reviewing several global and regional datasets that are useful for answering large-scale questions in river management. Looking specifically at river morphology, available datasets encompass the definition of river networks (Lehner et al. 2008; Yamazaki et al. 2019), the estimation of flow values (Barbarossa et al. 2018), the hydro-environmental classification of rivers (Ouellet Dallaire et al. 2019), river geometries and characteristics (Altenau et al. 2021) and soil erosion (Borrelli et al. 2017; Bezak et al. 2024), as well as information on bank erosion and deposition along rivers (Langhorst and Pavelsky 2023). Additionally, instream infrastructure, such as dam structures or barriers, is mapped and available as global datasets (Mulligan et al. 2020; Whittemore et al. 2020; Yang et al. 2022).

Similarly, global models have been developed to answer questions on global sediment budgets and to provide sediment transport information for data-scarce regions. The WBMsed model originally provides estimates of water discharge and suspended sediment flux and their dynamics (Cohen et al. 2013; Cohen et al. 2014) as well as their sensitivity to climate change (Moragoda and Cohen 2020). The model was recently enhanced to also incorporate bedload estimates (Cohen et al. 2022) but generally neglects sediment connectivity. On the other hand, the MOSART-sediment model (Li et al. 2022a) calculates sediment transport across the catchment and further accounts for soil erosion, reservoir operation and trapping. It is, however, currently limited to simulating suspended sediment transport in the US due to its dependence on numerous high-quality input data, especially for river geometry, median grain size, and river slope. The global sediment dynamic model (Hatono and Yoshimura 2020) accounts for both suspended and bedload, calculating sediment transport, erosion, and deposition, with a focus on clay, silt, and sand.

Despite the large number of global datasets on river characteristics, we still lack detailed information on river morphology or comprehensive sediment transport data to guide sustainable hydropower development. Another point is that these datasets, often developed from satellite information, do not include smaller rivers, such as those with widths below 30 m in the SWORD dataset (Altenau et al. 2021). The established global models, on the other hand, encompass quantitative information on sediment transport and its dynamics, but are also often limited in the resolution of the river network representation due to their global scope and often neglect sediment connectivity (Cohen et al. 2022). Due to these limitations, together with the frequent focus on suspended load or fine sediment transport (Hatono and Yoshimura 2020; Li et al. 2022a) and the requirement for detailed input data (Li et al. 2022a), those approaches are inadequate for guiding river management and sustainable hydropower development, particularly in developing regions.

Another approach to model sediment transport, specifically developed to guide hydropower planning, is the network-scale sediment connectivity model CASCADE (Schmitt et al. 2016; Tangi et al. 2019). Originally applied to the Mekong basin, the CASCADE model is able to model large river basins and focuses on sediment connectivity (Schmitt et al. 2018b; Schmitt et al. 2018a; Schmitt et al. 2019) but was also used to differentiate channel patterns in the less anthropogenically altered catchment (Bizzi et al. 2021). In comparison to the models mentioned above, the CASCADE model explicitly accounts for sediment connectivity and incorporates transport of a range of different grain sizes. However, its application also requires detailed local data on river discharge, riverbed slope, and river width.

Hence, currently available datasets and models remain limited in their applicability for supporting sustainable hydropower development. We identify a need for datasets that can inform three key application domains: (1) hydropower site comparison, facilitating the prioritization and exclusion of potential sites, similar to Schmitt et al. (2018a) and Schmitt et al. (2019); (2) design and operational decision-making, including structural and operational measures as described by Kondolf et al. (2014) and Gabbud and Lane (2016); and (3) assessment of ecological and morphological impacts, such as riverbed coarsening or siltation, as well as the overall reduction in habitat heterogeneity (Hauer et al. 2018; Schmutz and Moog 2018).

To overcome the limitations of existing datasets and models while still providing robust information for sustainable hydropower development in the data-scarce region of Central Asia, our study establishes a comprehensive geospatial dataset that describes sediment transport and connectivity at high resolution, utilizing openly available data. Building on the conceptual foundation and strengths of the CASCADE model, we develop a modified version tailored to data-scarce environments, focusing on sediment connectivity and the probability of bedload transport. The specific objectives of our study are:

1. To present a modified CASCADE model using input data derived from openly available geospatial datasets and statistically variable input data, enabling its application in the data-scarce region of Central Asia.
2. To develop a geospatial dataset based on validated simulation results of our modified CASCADE model providing high-resolution, qualitative information on sediment transport and sediment connectivity required for sediment management to guide sustainable hydropower planning

## Material and Methods

### Study Area

**Fig. 1:**
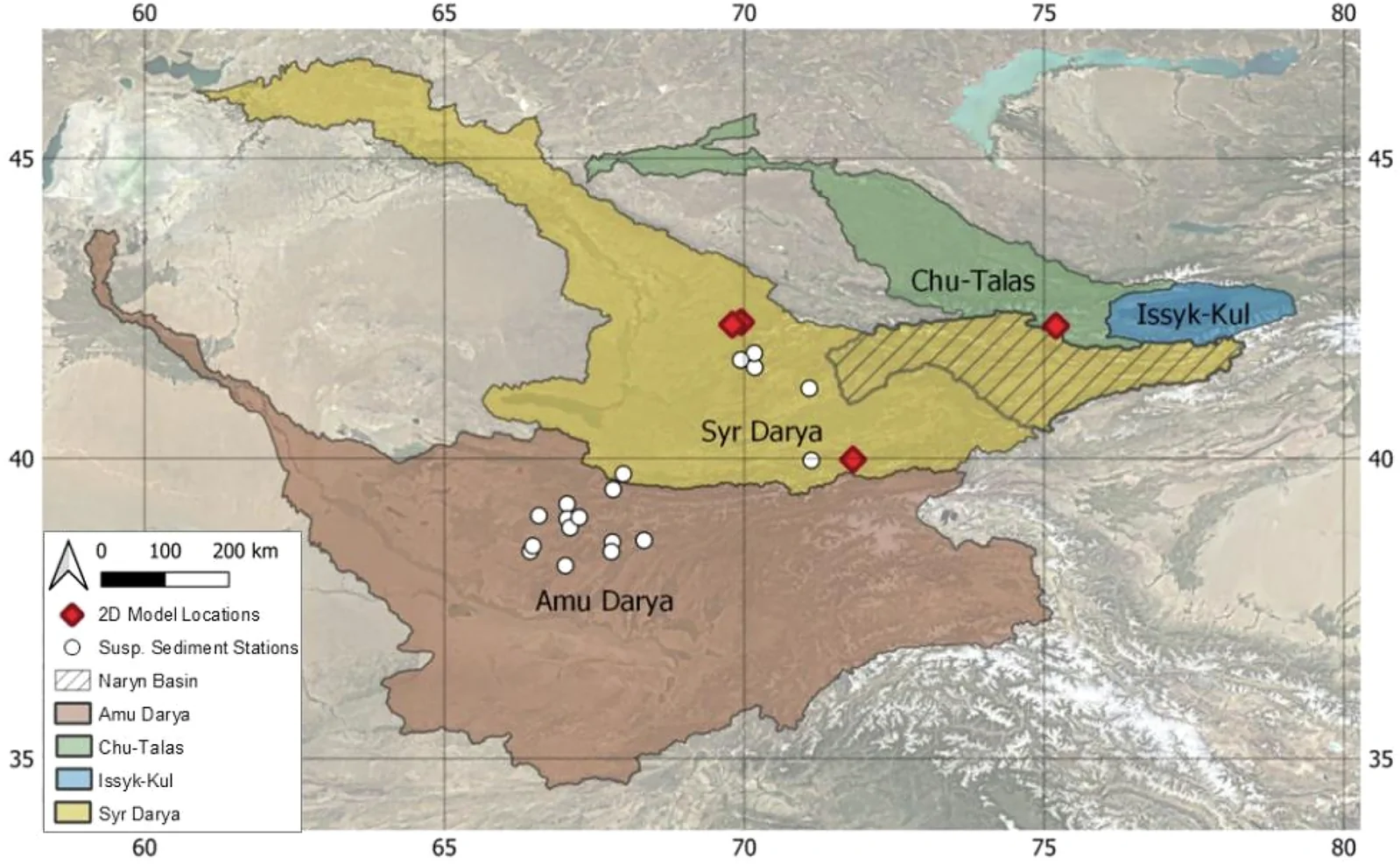
Study area showing the four basins included in the study across the region of Central Asia. Locations of the 2D models used for the result assessment are indicated by red diamonds. Locations of measured suspended sediment concentrations are indicated by white circles. The gray hatched region is the Naryn catchment (subbasin of Syr Darya), which is used for visualization purposes of the results. Coordinate Reference System (CRS): WGS 84 (EPSG:4326), Background: Google Earth imagery (Landsat and Copernicus data, composite)

Our study area covers the mountainous region of the five Central Asian countries, Uzbekistan, Tajikistan, Turkmenistan, Kyrgyzstan, and Kazakhstan, where hydropower, closely intertwined with irrigated agriculture, played a central role in industrial development during the 20th century and continues to be highly relevant today. In particular, the upstream countries Kyrgyzstan and Tajikistan remain strongly dependent on hydropower with more than 85% of their electricity production generated from hydroelectric plants (De Keyser et al. 2026b). We focus on the four main catchments of the region (following Marti et al. 2023b): Syr Darya, Amu Darya, Chu-Talas and Issyk-Kul (Fig. 1). Syr Darya and Amu Darya discharge into the Aral Sea, Issyk-Kul into Lake Issyk-Kul and Chu-Talas terminates in the Kazakh desert. These basins drain the entire Central Asian mountain range, comprising the Hindukush in the south, the Pamir and Gissar-Alay in the center, and the Tien Shan in the northeast. Discharge data for these catchments were recently processed and analyzed (CA-discharge, Marti et al. 2023a). The authors also classified the runoff regimes of the gauging stations as nivo-pluvial, nival, nivo-glacial or glacial-nival (Marti et al. 2023b), showing the strong influence of snow and glaciers in the high mountain range of Central Asia on the discharges in the basins. Data on sediment transport and hydromorphology are generally scarce, but for 18 of the stations of the CA-discharge dataset (Marti et al. 2023b) suspended sediment measurements provided as decadal monthly averages are available (Uzhydromet 2021, Fig. 1). These data offer a general overview of suspended sediment transport and its annual variability in the region.

### Input Data for the Modified CASCADE Model

**Fig. 2:**
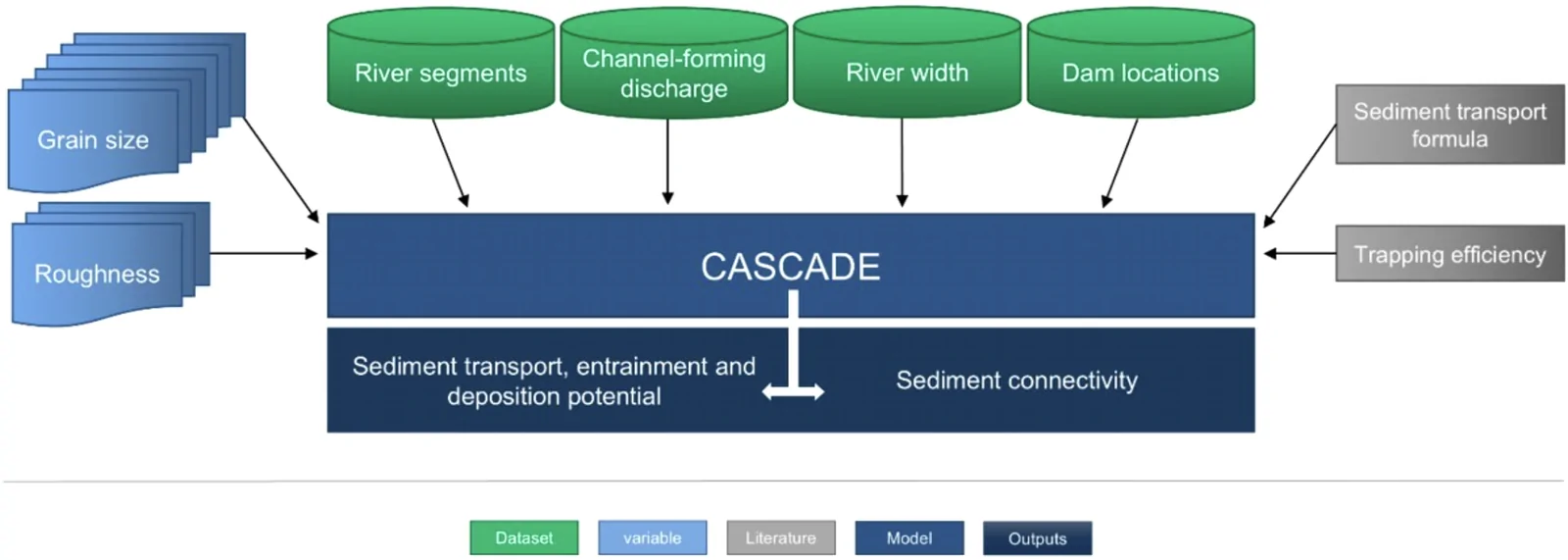
Model structure of the study, highlighting the different types of input data incorporated into the modified CASCADE model

CASCADE (CAtchment Sediment Connectivity And DElivery) models sediment transport by explicitly accounting for sediment transfer and deposition, thereby providing quantitative estimates of sediment connectivity at the network scale (Schmitt et al. 2016; Tangi et al. 2019). The CASCADE model in its traditional, non-dynamic form (Tangi et al. 2019) uses as input data a digital elevation model to obtain the river network and slope of river reaches, measured values for river width per reach, discharge percentiles obtained from gauging data, grain size distributions and Manning’s roughness values. Additional optional inputs are the location of sediment sources and barriers, with their sediment trapping efficiency (Tangi et al. 2019). To address our first objective, we adapted CASCADE to operate with large-scale, freely available geospatial datasets (Fig. 2), allowing basin-wide simulations in data-scarce regions. The modified model uses eight key inputs (Table 1). The sediment transport formula and trapping efficiencies follow previous CASCADE applications (Bizzi et al. 2021). All remaining datasets are described in the following subsections. In addition to the input data, the application of the CASCADE model within this study required adaptations in the simulation procedure, which are described in Section “Simulation procedure of the modified CASCADE model”.

**Table 1:** Overview and description of the input data

Input data	Type	Description	Processing	Reference
River segments	Dataset	Location and slope of flow paths	Flow network delineated from Lehner et al. (2008) and segmented into 1 km segments	HydroSHEDS (Lehner et al. 2008)
Channel-forming discharge	Dataset	Discharge value representing a channel-forming discharge at each river segment	Calculated as 5% percentile using the mean discharge obtained from Flo1K using a correlation established using the CA-discharge dataset	Flo1K dataset: Barbarossa et al. (2018)CA-discharge dataset: Marti et al. (2023a)
River width	Dataset	River width value corresponding to the channel-forming discharge value	Extracted from satellite images using Google Earth Engine (GEE) for images representing the month of the expected peak discharge obtained from the CA-discharge dataset, and using the RiVWidthCloud algorithm adapted to work on Sentinel-2 data	RivWidthCloud algorithm: Yang et al. (2020)CA-discharge dataset: Marti et al. (2023a)
Dam locations	Dataset	Location of dams in the river		GROD dataset Yang et al. (2022)
Grain sizes	Variable	Sediment grain sizes expressed as D_16_, D₅₀, D_84_	D_50_ in the range of fine silt to boulders, with changing values for grain size sorting to calculate D_16_ and D_84_ accordingly	Method following: Schmitt et al. (2018b)
Roughness	Variable	Roughness value of the riverbed used in hydraulic calculations	Set to range from 0.02 to 0.05 to represent smooth to rough rivers	/
Sediment transport formula	Literature	Transport capacity formula selected to calculate the potential sediment transport	Set to Parker & Klingeman formula (Parker and Klingeman 1982)	Method following: Bizzi et al. (2021)
Trapping efficiency	Literature	Proportion of sediment trapped behind barriers for each sediment class	Set to 99% for cobble, 90% for gravel, 70% for sand, 60% for silt	Method following the example dataset (Vjosa River) provided in the CASCADE software package (EI Team 2021)

#### River segments

A flow network for the region was delineated from the hydrologically conditioned HydroSHEDS Digital Elevation Model at 3-arcsecond resolution (Lehner et al. 2008). The conditioning of the original Shuttle Radar Topography Mission (SRTM) dataset included sink and void filling as well as stream burning to ensure a continuous river network over large areas. Streams of order one were excluded due to their predominantly ephemeral character (Hansen 2001). The resulting river network was divided into 1-km segments, which formed the basis for all subsequent analyses.

#### Channel-forming discharge

Bedload transport is mainly associated with high-flow events, when the shear stress exceeds the critical threshold of particle mobilization. To investigate bedload transport, we needed to select a corresponding discharge value for which sediment transport is expected to occur. Literature refers to this discharge value as “effective” or “dominant” discharge and it is often linked to the bankfull discharge (Church and Ferguson 2015). According to Leopold (1995), bankfull discharge and thus significant sediment transport typically correspond to events with a return period of roughly 1 to 2.5 years. With the assumption that sediment transport influences channel form annually during high-flow events, we developed a correlation to extract discharge percentiles from the Flo1K dataset (Barbarossa et al. 2018) for each river reach. These discharge percentiles are calculated by multiplying a correction factor by the mean monthly value from the Flo1K dataset (Barbarossa et al. 2018). The correction factor is a function of the seasonal variation index, which is obtained by correlating the percentiles of the time series from the CA-discharge dataset (Marti et al. 2023a) to the seasonal variation index. Based on this approach, we determined Q5, defined as the discharge that is exceeded 5% of the time based on mean monthly flow observations. Further information is presented in Supplement 1.

#### River width

We obtained the river width information by modifying the RivWidthCloud algorithm (Yang et al. 2020) in Google Earth Engine (Gorelick et al. 2017) to work with the Sentinel-2 mission instead of the Landsat mission. The higher spatial resolution of Sentinel-2 allowed us to extract width information for smaller channels than is possible with Landsat. It was necessary to select river-width information when the discharge is highest during the year, corresponding to the channel-forming discharge values (see Supplement 2 for more information). For each Sentinel-2 tile covering our four river basins, we hence obtained an associated month for which the river width is expected to be at its maximum. Using these tile-month relations in our edited RivWidthCloud algorithm, we extracted a series of width data points for each catchment. Each measurement point was linked to the nearest 1-km river segment, and the median width value for each segment was selected from all available width measurements. For reaches where no width information could be derived by processing the Sentinel-2 data, we calculated the river width using a proportional relation of river width and discharge taken from a nearby river reach with comparable discharge conditions.

#### Dam locations

The locations of dam structures in the Central Asian rivers are taken from the GROD dataset (Yang et al. 2022). Each GROD data point was assigned to the nearest river reach based on minimum distance. Each dam is assigned a trapping efficiency (Table 1), which represents the effect of river barriers on sediment transport and connectivity. The trapping efficiency defines the proportion of the sediment load, for a given grain size, that is retained at the barrier. Consequently, the upstream sediment load is reduced by this proportion, with the retained material deposited within the respective reach, thereby also reducing sediment flux to downstream reaches.

#### Sediment grain sizes and roughness

Information on sediment grain size distribution and riverbed roughness is unavailable for the Central Asian catchments at the resolution of our river segments (1 km). To account for this data scarcity, we prepared a broad range of grain size distributions and roughness values as input parameters for our modified CASCADE model. The synthetic grain sizes were generated for five sediment classes: silt, sand, gravel, cobble, and boulders. Following ISO (2017), the classes silt, sand, and gravel were further subdivided into fine, medium, and coarse (Table 1). This results in a total of eleven sediment classes. For each class, ten evenly spaced D₅₀ values were assigned between the class-specific minimum and maximum grain sizes, yielding 110 distinct D₅₀ values. For each D₅₀ value, three corresponding D₁₆ and D₈₄ values were calculated to generate well-sorted, moderately sorted, and poorly sorted grain-size distributions. In total, this results in 330 synthetic grain-size distributions. Differences in sorting were achieved by varying the geometric standard deviation to 1.3, 1.6, and 2.0 for well-sorted, moderately sorted, and poorly sorted material, respectively (Inman 1952; Blott and Pye 2001). The riverbed roughness was assumed to be in the range of 0.02 to 0.05 with an increment of 0.005 to represent smooth to very rough riverbeds.

### Simulation Procedure of the Modified CASCADE Model

We ran the CASCADE model using the described input data in the four Central Asian catchments (Fig. 1), with the following modifications to the standard model. These modifications serve, on the one hand, to adapt the model to the reduced input data, particularly the absence of flow percentiles and measured grain-size distribution, and, on the other hand, to produce results that can be interpreted directly in terms of the risks associated with sediment transport and the potential effects of its alteration. Therefore, we used only one channel-forming discharge, along with the associated river width, as described above, instead of several discharge percentiles. Secondly, we simulated the sediment transport capacity for each of the 330 grain size distributions combined with the seven roughness values, resulting in a total of 2310 simulation runs per catchment. The results of all simulations were then aggregated to determine the probability of transport, deposition, and entrainment for each sediment class, calculated as the proportion of runs in which each process occurred in each river reach. Transport capacity is computed independently for each reach, while entrainment and deposition are derived from the comparison between incoming sediment load and transport capacity. CASCADE further provides a connectivity matrix indicating which upstream reaches contribute sediment to downstream reaches. A reach is considered connected when sediment from an upstream reach can be transported through the target reach, thereby contributing to downstream sediment transfer. Based on this, we quantified connectivity by counting the number of upstream reaches contributing sediment to each reach and defined a connectivity index as the ratio of connected upstream reaches to the total number of upstream reaches.

For computational purposes, we divided each catchment, except Issyk-Kul, into sub-catchments using level 5 basins of Lehner et al. (2008). However, the sediment transfer between sub-catchments was maintained by exporting the sediment transport information of the outlet and importing it as an external sediment source into the upstream reach of the downstream sub-catchment. These modifications of the CASCADE model enabled us to address the second objective of our study by generating simulation results that were subsequently aggregated into a geospatial dataset, providing information suitable for supporting sediment management and sustainable hydropower planning.

### Data Assessment

#### Assessment of input data

In our modified CASCADE model, we utilize existing geospatial datasets for defining river segments, discharge, and the location of barriers (Table 1). The river width data is the only newly created input dataset in this study. To assess the accuracy of this newly created dataset, we measured river width at randomly distributed points in all four catchments using high-resolution aerial images available on Google Earth. Additionally, a visual assessment of the input data for river slope, channel-forming discharge and river width is conducted to verify the plausibility of the data.

#### Assessment of results of the modified CASCADE model

Because measured data on total sediment transport are scarce, and our model does not provide specific sediment fluxes, direct verification of the CASCADE simulation results with measured data is not possible. The available suspended sediment measurements at the 18 stations provide, however, valuable insights into where the fine sediment fractions are regularly transported. Therefore, we extracted the modeled sediment transport probabilities for sand and silt at the stations where suspended sediment measurements were taken and assessed whether the model produced high transport probabilities for these grain fractions.

More importantly, we conducted a result assessment as an internal consistency check to assess whether the modified CASCADE model produces results within an expected and reasonable range. For this plausibility assessment, we compared the modified CASCADE results to the sediment transport probabilities obtained from 2D hydraulic simulations for four sites within the study area (Fig. 1). The 2D models are further described in Table 2. The models have a much higher resolution regarding river geometry and hydraulic values, providing a more accurate representation of the hydraulic and corresponding hydromorphologic conditions. All models were hydraulically calibrated by adjusting the roughness values to match measured water level elevations, following the steps described in Hayes et al. (2025).

**Table 2:** Description of 2D models used in the plausibility assessment

Site	Catchment	Length	Slope 2D model	Slope CASCADE	Bankfull discharge	Element size	Number of cross-sections used for comparison
Badam	Syr Darya	1120 m	0.0074	0.0077	27 m³/s	0.6–1.8 m	15
Sairam	Syr Darya	880 m	0.0106	0.0096	54 m³/s	0.5–1 m	15
Shakimardan	Syr Darya	3100 m	0.0331	0.0786	17 m³/s	0.25 m	30
Suyok	Chu-Talas	280 m	0.0160	0.0187	37 m³/s	0.25 m	15

**Fig. 3:**
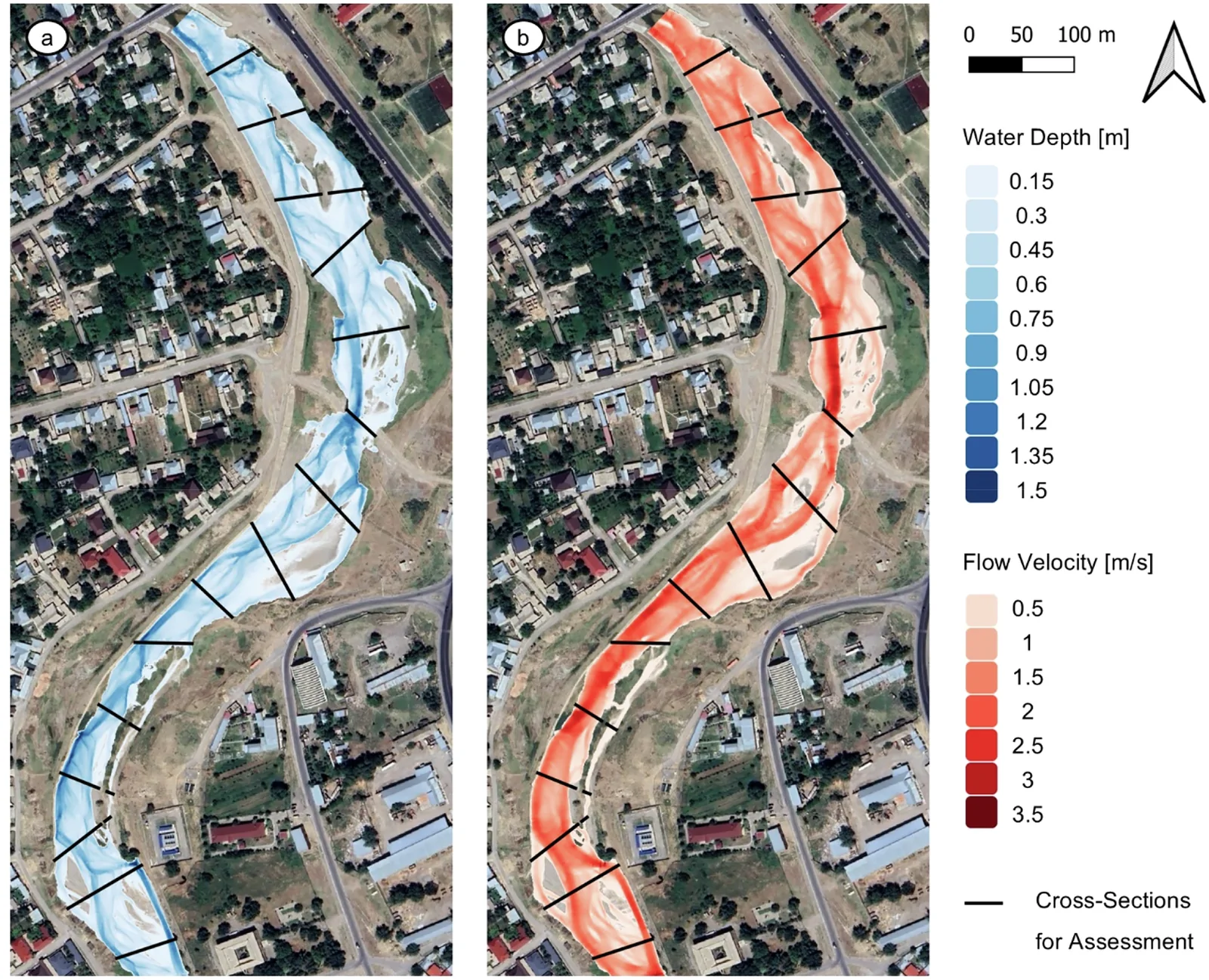
Example for Badam site: **a** simulated water depths and **b** depth-averaged flow velocity for a discharge value of 27 m³/s; black lines indicate the locations of the 15 cross-sections used to extract hydraulic parameters for the plausibility assessment. Background: Google Earth imagery 2025 Airbus

Hydraulic conditions for the simulated channel-forming discharge at several cross-sections were extracted within the 2D model domains and inserted into the sediment transport capacity formula, consistent with the CASCADE model (Parker and Klingeman 1982). This calculation was repeated using the previously defined 330 grain size distributions. Figure 3 shows the modeled water depth and flow velocity, as well as the cross-sections used in the plausibility assessment for the Badam site.

#### Sediment transport dataset

The final result of this study is a geospatial dataset comprising the modified CASCADE simulation results as geospatial map layers. The dataset comprises all 1-km-long river segments for the four basins included in the study area: Amu Darya, Syr Darya, Chu-Talas and Issyk-Kul. Each river segment contains values for sediment transport probabilities, deposition probabilities, and entrainment probabilities for each grain size as well as the sediment connectivity index. All values, probabilities and connectivity index are in the range of zero to one, which facilitates the visualization and interpretation of the data. We propose interpreting the probability values as follows: very low (0–0.2), low (0.2–0.4), medium (0.4–0.6), high (0.6–0.8), and very high (0.8–1).

## Results

### Assessment of Input Data

**Fig. 4:**
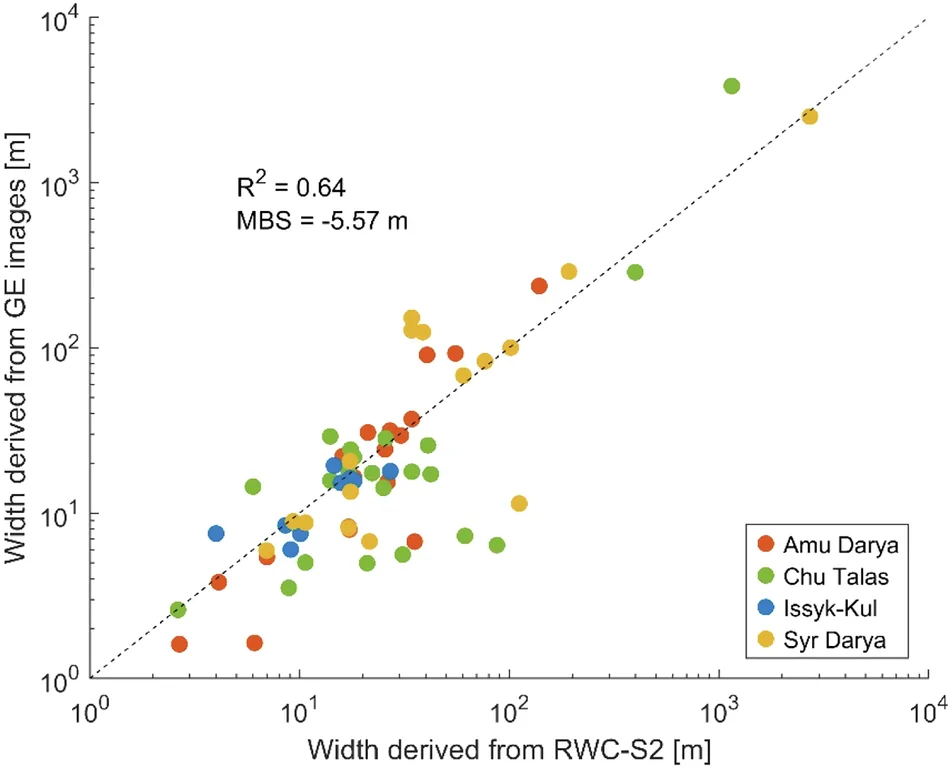
Accuracy assessment of river widths obtained from the modified RivWidthCloud algorithm using Sentinel-2 data (RWC-S2) against measured river width from Google Earth images (GE) at randomly selected points in each of the four catchments. Black dotted line indicates a perfect agreement. R² is the coefficient of determination, and MBS is the mean bias.

Figure 4 compares manually measured data from high-resolution Google Earth (GE) imagery with data produced by the modified RivWidthCloud algorithm (RWC-S2). The calculated RWC-S2 river width estimates exhibit scatter around the line of perfect agreement (dotted line) and a general tendency toward underestimation, as indicated by the mean bias (MBS) of ~6 m. The highest agreement is observed in the Issyk-Kul catchment, while the lowest occurs in the Chu-Talas catchment.

**Fig. 5:**
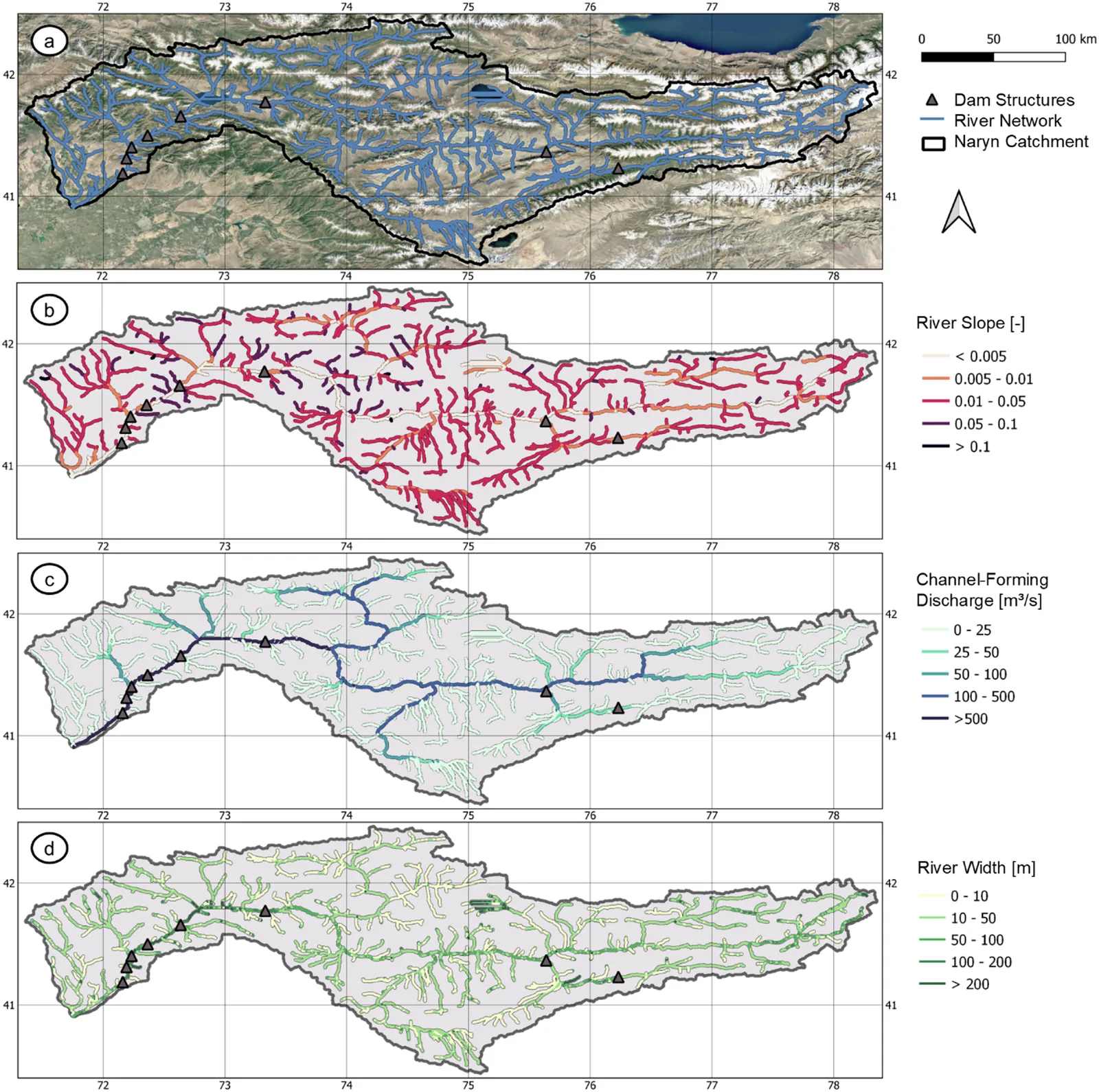
Prepared input data for the example of the Naryn catchment: **a** river segments located within the catchment with the catchment discharging from east to south-west, input data sets (**b**) river slope [-], **c** channel-forming discharge [m³/s], and **d** river width [m]. Black triangles represent the location of dam structures taken from GROD dataset (Yang et al. 2022). Coordinate Reference System (CRS): WGS 84 (EPSG:4326), Background of (**a**): Google Earth imagery (Landsat and Copernicus data, composite)

Figure 5 illustrates the prepared input datasets for the Naryn catchment, a sub-catchment of the Syr Darya River (compare Fig. 1). The Naryn catchment is a mountainous area, as shown in the satellite image in Fig. 5a. A clear longitudinal trend is evident, characterized by decreasing slope (Fig. 5b) and increasing channel-forming discharge (Fig. 5c) in the downstream direction. Locally reduced slope values can also be observed upstream of dam structures, although overall slope remains generally high due to the mountainous character of the Naryn catchment. River width values increase with increasing discharge (Fig. 5d) but show greater spatial variability than slope and discharge due to local topographic controls, such as lakes and valley confinements.

### Assessment of Results of the Modified CASCADE Model

**Fig. 6:**
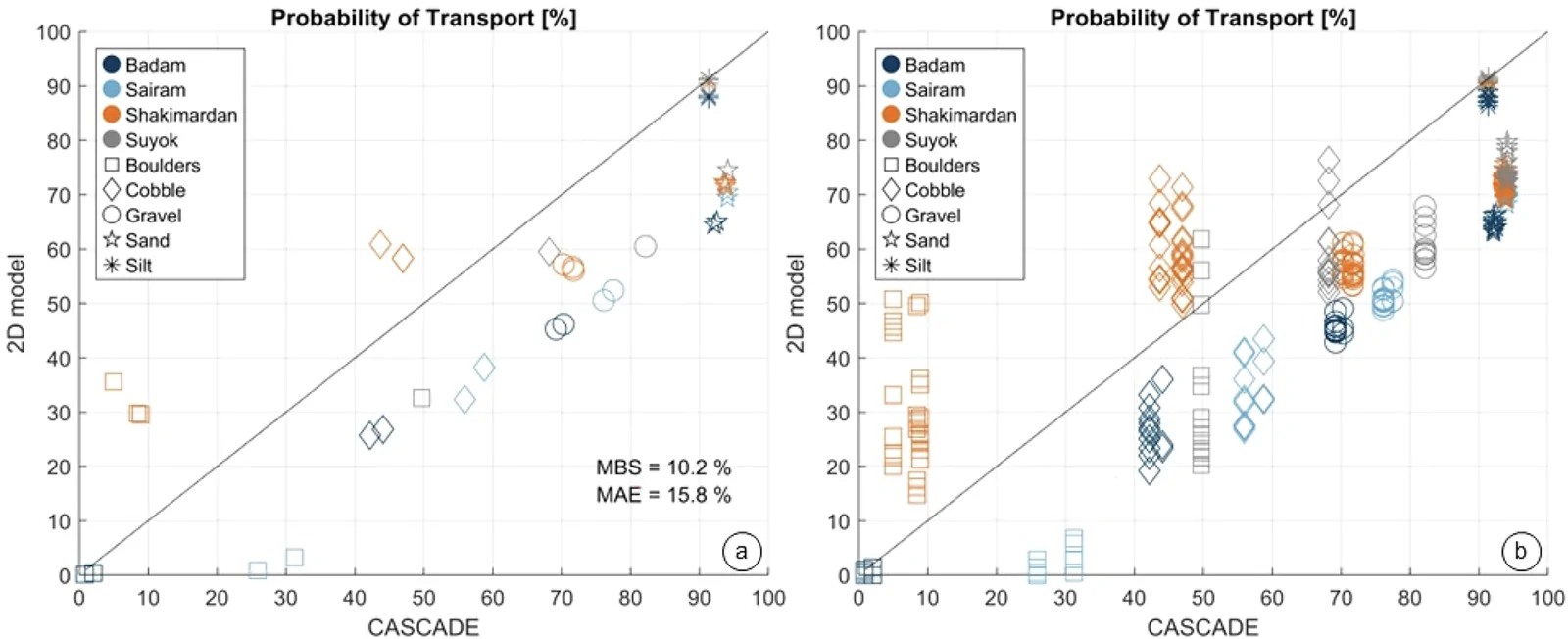
Accuracy assessment of CASCADE simulation results compared to results obtained from the 2D models; **a** mean values averaged over all cross-sections for each river segment covered by the 2D models; **b** values of each cross-section in the 2D model against the associated values per river segment covered by the 2D models. Black line indicates a perfect agreement. MBS is the mean bias, and MAE is the mean error.

Figure 6 compares the transport probabilities for all five grain size classes obtained by the modified CASCADE model with the values obtained by analyzing the results of the 2D models. Figure 6a displays the probabilities averaged over all cross-sections for each river segment covered by the 2D models, whereas Fig. 6b includes the values for all available cross-sections of each 2D model site. Both models, CASCADE and each 2D hydrodynamic model, calculate an increase in transport probability for decreasing sediment grain sizes. The modified CASCADE model generally overestimates the probabilities, with a mean error (MAE) of ~16% and a mean bias (MBS) of 10%. This overestimation is visible for three of the four 2D model sites, Badam, Sairam and Suyok. The transport probability values obtained by CASCADE for boulders and cobbles at Shakimardan site, on the other hand, show an underestimation compared to the results of the corresponding 2D model. Figure 6b visualizes the variances of the results between the cross-sections of each 2D model site, which results from local variances in water depth and flow velocity. Hence, it can be observed that this variability within one site is in a similar range to the magnitude of the mean error.

In addition, all 18 suspended sediment stations showed measured values above 100 g/s, averaged over three decades, for the month of the highest sediment flux, indicating regular sediment transport of fine sediments. The modeled transport probabilities for silt and sand at these stations showed good agreement with the measured data, with values ranging from 76% to 94%, and mean values of 90% and 94%, respectively, for silt and sand.

### Sediment Transport Dataset: Exemplary for the Naryn Catchment

Our study delivers a dataset providing high-resolution, qualitative information on sediment transport and sediment connectivity, which covers the entire Central Asian region, as illustrated in Fig. 1. This dataset is freely available for download and is integrated in the hydropower decision support system (HydroPlan-CA, De Keyser et al. 2026c).

**Fig. 7:**
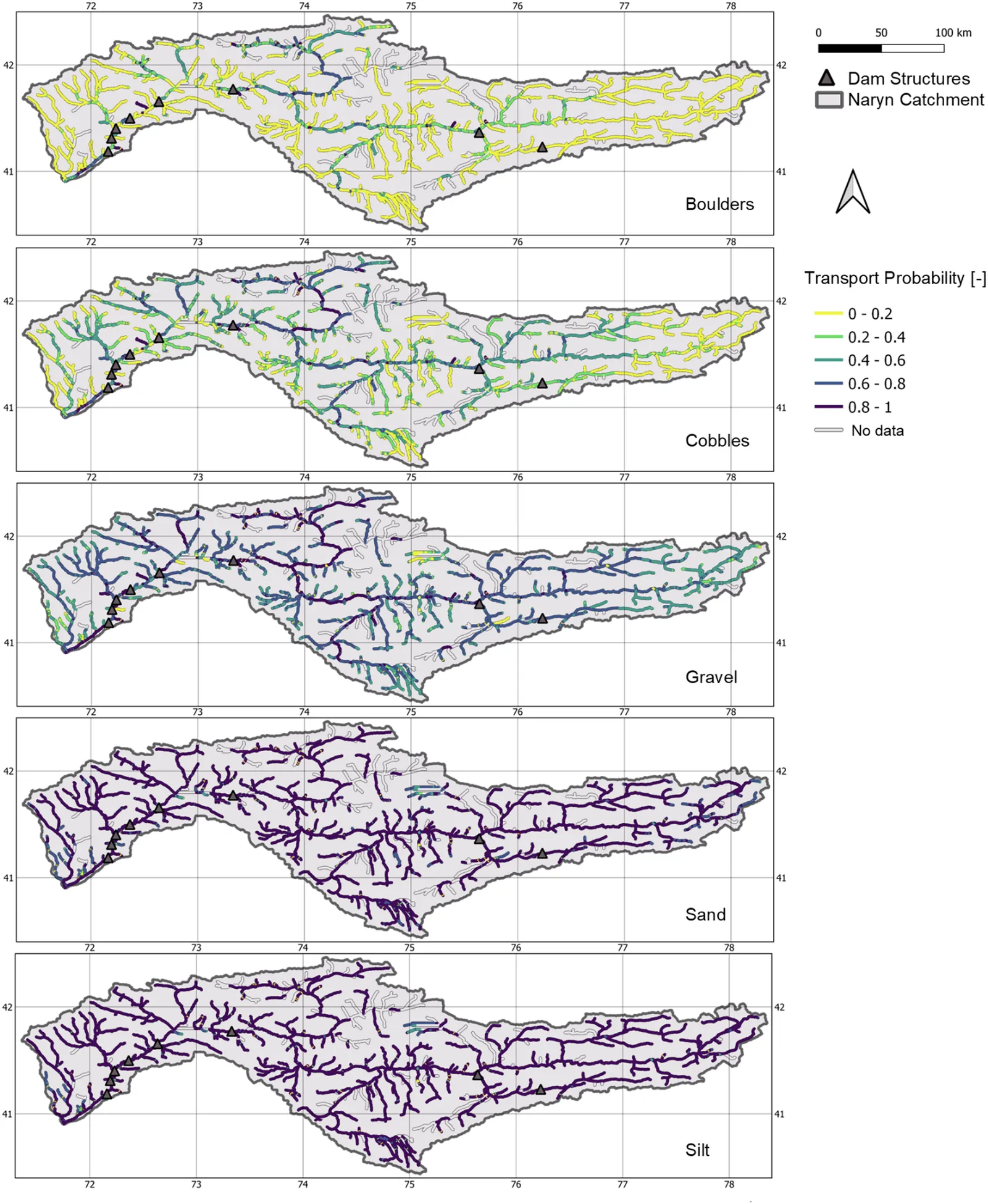
Maps of sediment transport potential for each grain size class in the Naryn catchment. Coordinate Reference System (CRS): WGS 84 (EPSG:4326)

For visualization purposes, we provide the dataset for the Naryn catchment. Figure 7 shows the sediment transport probabilities for each sediment class in the Naryn catchment with higher probabilities for finer sediment sizes as expected. Boulders are expected to be transported only in the main river with low to high probabilities, where the discharge is highest in the catchment (Fig. 5). Cobbles are likely to be transported in the entire main river (medium to very high probabilities), whereas gravel is mobilized in almost all river segments, including small tributaries, with medium to very high probabilities. Sand and silt show high to very high probabilities of transport throughout the catchment. Hence, for the Naryn catchment, the results indicate that we can expect high sediment mobility, especially of gravel, sand and silt, likely linked to substantial sediment fluxes.

**Fig. 8:**
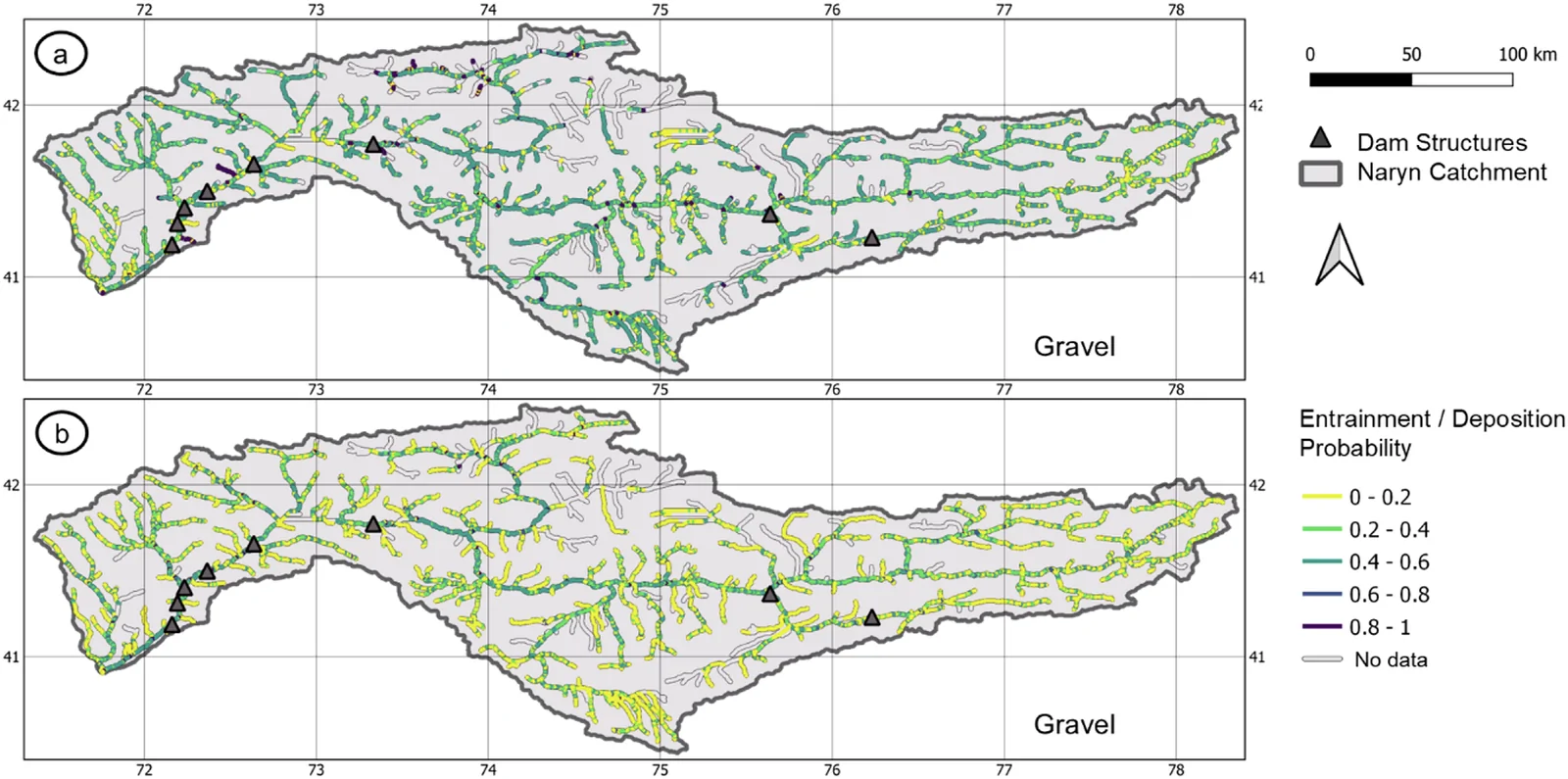
Maps of (**a**) sediment entrainment and **b** deposition probability shown exemplarily for gravel in the Naryn catchment. Coordinate Reference System (CRS): WGS 84 (EPSG:4326)

Figure 8 shows the entrainment and deposition probabilities, exemplary for the sediment class of gravel. There is a higher variability in the values between the river segments compared to the transport probability of gravel in Fig. 7. This variability arises from the upstream–downstream comparison used to derive these values, meaning that entrainment and deposition depend not only on the transport capacity of the current reach but also on the incoming sediment load. For the deposition probability, there is a pattern of increasing probability from very low to medium when moving downstream. Entrainment, on the other hand, does not show a spatial pattern and is mostly in the range of low to medium probabilities. Generally, we can observe that Naryn catchment, due to its mountainous character, is more prone to entrainment than to deposition of gravel. These low deposition probabilities, especially in the steep tributaries, show that mobilized gravel is transported further downstream to the main river. In the main river, deposition might occur but coincides with high transport probabilities (Fig. 7) and medium entrainment probabilities, which indicate highly active riverbeds and frequent hydromorphological adjustments.

**Fig. 9:**
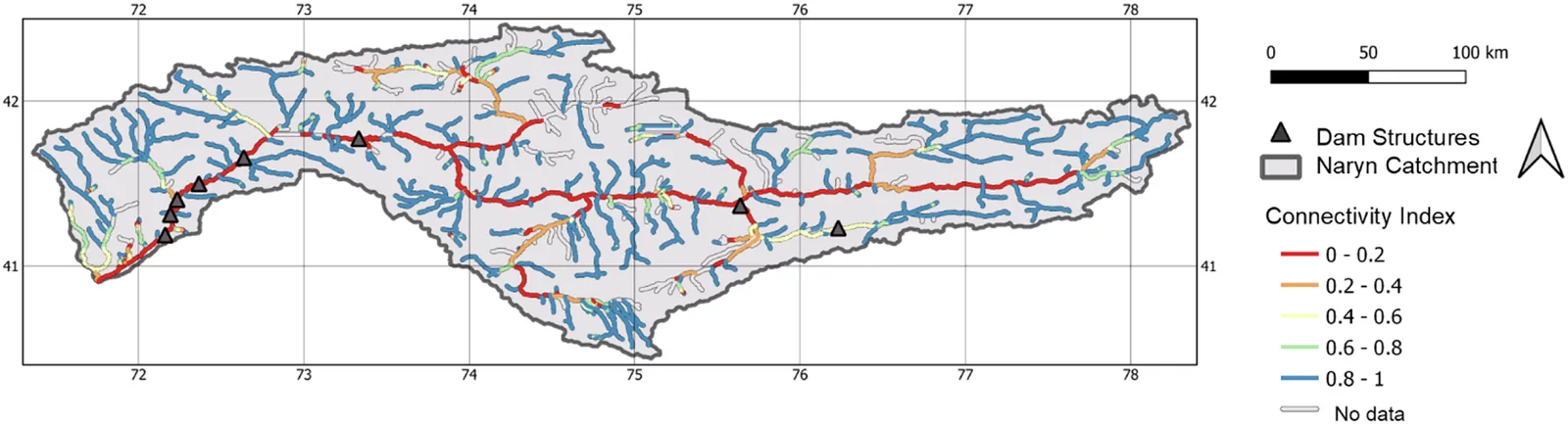
Map of sediment connectivity index in the Naryn catchment. Coordinate Reference System (CRS): WGS 84 (EPSG:4326)

Figure 9 shows the sediment connectivity index calculated for the Naryn catchment, which is independent of the grain size. According to the modeling results, the connectivity is expected to be highest in the tributaries, where upstream river segments are able to transfer the incoming sediment load. The main river, on the other hand, has very low connectivity indices, showing that as the upstream catchment increases, river segments become less connected to their upstream reaches. As shown in Fig. 8 exemplarily for gravel, deposition can occur in the main river, whereas it is less likely in the tributaries. A local deposition of sediments in the main river can interrupt sediment transfer and lead to a loss of connectivity to upstream reaches. Obstructions, such as dams, further increase the risk of deposition and hence additionally reduce the connectivity in the main river.

## Discussion

### Model Development, Evaluation and Uncertainty Assessment

We successfully modified the CASCADE model to enable its application in the data-scarce region of Central Asia, where information on sediment transport is crucial for sustainable hydropower development. These modifications rely on the use of openly available geospatial datasets and algorithms to derive discharge, river slope, and river width, as well as the statistical variation of roughness values and sediment grain sizes. These adjustments can reduce the reliance on locally measured data and may enhance the model’s transferability to regions with limited field observations, although the approach can still be data-, time-, and computation-intensive. An accuracy assessment of the river width data yielded good results, despite some differences compared to manually measured widths obtained from Google Earth. Our modified CASCADE model produces information on expected morphological processes in the form of transport, deposition, and entrainment probabilities for five different sediment grain sizes: silt, sand, gravel, cobble, and boulders. Additionally, sediment connectivity is calculated to inform about upstream sediment supply. A comparison of the modified CASCADE results with sediment transport probabilities extracted from 2D numerical hydraulic simulations at four sites reveals that CASCADE generally predicts higher probabilities but follows the same physically expected pattern of increasing probabilities with decreasing grain sizes. Since the input data assessment and simulation result assessment indicated good results, we applied the model to the four main river basins of our study: Amu Darya, Syr Darya, Chu-Talas and Issyk-Kul. By aggregating these simulation results into maps, we obtained, for the first time, a geospatial dataset with a 1-km resolution, providing information on sediment transport probabilities for five sediment grain size classes that covers the main river basins of Central Asia. The representation of the data in the form of probabilities or connectivity indices facilitates the use of the dataset for sediment management in relation to sustainable hydropower development (see Section “A guidance tool for each key development stage in sustainable hydropower development”).

Model assumptions, the 1-km resolution and uncertainties in the input data may have affected the results of the modified CASCADE. To account for missing grain-size distributions, we defined a broad range of possible distributions and simulated their transport across the entire study area. This assumption requires substantial computational effort and may limit its applicability as a rapid assessment method in other data-scarce regions. Where expert knowledge or measured grain-size distributions are available, the approach could be simplified by focusing on a single, representative distribution. However, we deliberately avoided selecting a single grain-size distribution to, firstly, capture the wide diversity of river systems within the study area, ranging from coarse sediment transport in mountainous rivers to predominantly fine sediment transport in lowland systems. Secondly, this strategy ensures a consistent and representative dataset across the entire Central Asian region. The influence of the resolution can be seen when visualizing the transport probabilities calculated for the different cross-sections, where the results of the 2D models were extracted (Fig. 6). The variability in hydraulic conditions between the cross-sections results consequently in different sediment transport probabilities. These local changes in hydraulic conditions are not considered when aggregating the results in 1-km segments. For basin-wide sediment management, this 1-km resolution is, however, sufficient and finer than what global models have provided so far (Hatono and Yoshimura 2020; Cohen et al. 2022; Li et al. 2022a). Uncertainties in the input dataset for river width arise from the use of satellite data to detect surface water within the RivWidthCloud algorithms (Yang et al. 2020). Therefore, we assume that the highest uncertainties within our study arise from the data on river width. Looking analytically at the sediment transport formula used in this study, the Parker-Klingeman formula (Parker and Klingeman 1982) as implemented by Bizzi et al. (2021), we recognize that river width, in comparison to river slope and discharge, has the lowest influence on sediment transport capacity. The low sensitivity of the formula to variations in river width, combined with the absence of more accurate datasets, justifies the use of the river width dataset created within this study.

Regarding the results, we anticipate that the connectivity index is the most sensitive to uncertainties in input data and model assumptions. Its value depends on the input data of both the current reach and the upstream reaches. If inaccuracies in the input data lead to an underestimation of transport capacity, incoming sediment from upstream may not be transported further downstream, resulting in a disruption of connectivity. In addition, missing input data, particularly a lack of identified river width, can strongly affect connectivity, potentially causing local interruptions. Furthermore, as the connectivity index is a relative measure comparing the number of morphologically connected upstream reaches to the total number of upstream reaches, high connectivity values in downstream parts of the catchment become increasingly difficult to achieve if connectivity is interrupted locally. Despite these uncertainties, the connectivity index provides valuable insights into the spatial structure of sediment transfer across the river network. While individual values may be sensitive to local data limitations, the index captures overarching patterns of upstream-downstream connectivity that cannot be derived from reach-scale transport characteristics alone. The combined analysis of the connectivity together with the other results, transport, entrainment and deposition probability, captures both local and network-scale processes and thereby supports more informed and sustainable hydropower planning.

### A Guidance Tool for Each Key Development Stage in Sustainable Hydropower Development

Hydropower plants significantly alter sediment transport and river morphology, with these impacts being particularly strong in sediment-rich alpine regions (Gabbud and Lane 2016). Despite considerable untapped hydropower potential in Central Asia (Azimov and Avezova 2022; De Keyser et al. 2026a; De Keyser et al. 2026b), the lack of sediment-related information limits the ability to assess geomorphological impacts and the sustainability of potential hydropower projects. Our developed geospatial dataset comprises three complementary layers of information. The probability of transport for each sediment size indicates whether a river reach has the capacity to transport that sediment at a representative (bed-forming) discharge. It therefore provides insight into expected sediment fluxes and dominant, mobilized sediment sizes within a given reach. The probability of entrainment or deposition reflects the relative transport capacity of a reach compared to its upstream reach. If the upstream transport capacity exceeds that of the downstream reach, deposition is likely to occur; conversely, higher transport capacity in the downstream reach may lead to additional entrainment. These layers thus indicate whether a reach is prone to erosion or deposition. The connectivity index quantifies the degree to which upstream sediment is transferred through the river network, thereby capturing upstream–downstream interactions and sediment continuity.

Below we discuss specifically how our dataset can be used in each of the three identified key application domains: (1) hydropower site comparison, (2) design and operational decision-making, and (3) assessment of ecological and morphological impacts. We further illustrate the application of this dataset for two sites, Suyok and Shakimardan (Table 2). Both sites are located in mountainous regions but differ in discharge characteristics and slope (Table 2). The specific values for both sites, along with short descriptions, can be found in Supplement 3.

1. Hydropower site comparison: the dataset enables a consistent comparison of potential hydropower sites across the Central Asian region. Reaches with low transport probabilities may be favorable from an engineering perspective, as lower morphological activity can facilitate infrastructure stability and operation. However, as hydropower development typically reduces channel gradients and enhances deposition, sites that are already deposition-prone may be particularly susceptible to reservoir siltation. Similarly, reaches with low connectivity may be advantageous from an operational perspective, while highly connected reaches are critical for maintaining sediment continuity and ecosystem functioning. This highlights an inherent trade-off between engineering feasibility and environmental sustainability. When comparing hydropower development at both example sites, structural stability and the management of coarse sediment fluxes are more critical at the Suyok site, as coarse particles (boulders and cobbles) dominate sediment transport. In contrast, sediment dynamics at Shakimardan are characterized by the mobilization and deposition of finer material (gravel to silt). However, Shakimardan exhibits strong connectivity to upstream reaches, emphasizing the importance of maintaining sediment continuity at this site.
2. Design and operational decision-making: the dataset provides guidance on the suitability of different hydropower configurations and the need for mitigation measures. High transport probabilities indicate increased sediment loads, requiring robust design and operational strategies, such as reinforced structures, sediment flushing or bypass systems, and desanders. These measures are particularly important in depositional or highly connected systems to maintain sediment continuity and reduce operational risks. At both example sites, appropriate design and operational configurations are required to maintain sediment continuity, including flushing or bypass systems, due to high to very high bedload transport probabilities and potential deposition across a range of sediment sizes. In addition, both sites show very high transport probabilities for fine sediments (sand and silt), indicating the need for sediment control measures, such as desanders, to protect turbine infrastructure.
3. Ecological and morphological impacts: the dataset supports the identification of processes that may be altered by hydropower development. Changes due to hydropower implementation such as reduced channel slope, altered discharge regimes, and impoundment can decrease transport capacity, increase deposition, and disrupt sediment connectivity. These processes may significantly impact river morphology and reduce habitat heterogeneity. Such ecological and morphological impacts are expected at both example sites, as high transport capacities, erosion and deposition patterns, and overall connectivity are likely to be affected by discharge alteration or impoundment linked to hydropower development. At the Shakimardan site, an increase in fine-sediment deposition is likely, as the reaches are already deposition-prone. At the Suyok site, hydropower development could reduce riverbed dynamics in comparison to the currently high mobility of the channel, characterized by elevated transport, erosion, and deposition probabilities. In both cases, a reduction in habitat heterogeneity and quality is likely, highlighting the need for mitigation and habitat restoration measures.

Individually or in combination across the three key application domains, the dataset supports and facilitates decision-making in hydropower planning, helping to identify economically viable sites while minimizing and mitigating negative impacts on riverine ecosystems. Hence, the dataset is intended as a decision-support tool to facilitate more informed and sustainable hydropower development in Central Asia rather than a prescriptive solution. The different data layers should be interpreted in combination, validated where possible, and complemented by site-specific data and expert judgment. Due to its spatial resolution (1 km), the dataset is not suitable for detailed design at individual sites but is particularly valuable during early-stage planning. It does not provide direct recommendations on where hydropower should be developed or avoided; instead, it offers a consistent and transparent basis for comparing sites and identifying potential risks. The dataset can be integrated with additional information (e.g. De Keyser et al. 2026b and De Keyser et al. 2026c) and can support tools, e.g., RESCON (Efthymiou et al. 2003), which require sediment-related inputs for economic and environmental assessments.

## Conclusions

This study presents a modified version of the CASCADE model, utilizing input data derived from openly available geospatial datasets and statistically variable input data, which enables its application in the data-scarce region of Central Asia. The modified CASCADE simulations then result in sediment transport probability information for the sediment grain sizes silt, sand, gravel, cobble and boulders. The resulting geospatial dataset provides the first high-resolution estimation of sediment transport probabilities and connectivity for Central Asia. It covers the basins of the Amu Darya, Syr Darya, Chu-Talas, and Issyk-Kul, providing valuable information for basin-scale sediment management and hydropower planning. Simulation results have been assessed against those of 2D numerical hydraulic simulations at four sites and show good agreement. The final dataset supports the decision-making process of hydropower planning within three key application domains: hydropower site comparison, design and operational decision-making, and assessment of ecological and morphological impacts. The practical applicability of the dataset is further demonstrated through two example sites, illustrating how the derived data can support these key application domains. While the dataset cannot substitute for detailed site-specific assessments, it provides a valuable tool for basin-scale sediment management and early-stage decision-making in the sustainable development of hydropower in Central Asia.

## Supplementary information

Supplementary information

## Data Availability

The dataset “CA_sediment” generated in this study is available at https://doi.org/10.5281/zenodo.17712636.

## References

[CR1] Altenau EH, Pavelsky TM, Durand MT, Yang X, Frasson RPdM, Bendezu L (2021) The Surface Water and Ocean Topography (SWOT) Mission River Database (SWORD): A Global River Network for Satellite Data Products. Water Resour Res 57 10.1029/2021WR030054.

[CR2] Azimov U, Avezova N (2022) Sustainable small-scale hydropower solutions in Central Asian countries for local and cross-border energy/water supply. Renew Sustain Energy Rev 167:112726. 10.1016/j.rser.2022.112726.

[CR3] Barbarossa V, Huijbregts MAJ, Beusen AHW, Beck HE, King H, Schipper AM (2018) FLO1K, global maps of mean, maximum and minimum annual streamflow at 1 km resolution from 1960 through 2015. Sci Data 5:180052. 10.1038/sdata.2018.52. PMC5870339 29583139

[CR4] Best J (2019) Anthropogenic stresses on the world’s big rivers. Nat Geosci 12:7–21. 10.1038/s41561-018-0262-x.

[CR5] Betz F, Rauschenberger J, Lauermann M, Cyffka B (2016) Using GIS and Remote Sensing for Assessing Riparian Ecosystems along the Naryn River, Kyrgyzstan. Int J Geoinformatics 12:25–30

[CR6] Bezak N, Borrelli P, Mikoš M, Jemec Auflič M, Panagos P (2024) Towards multi-model soil erosion modelling: An evaluation of the erosion potential method (EPM) for global soil erosion assessments. CATENA 234:107596. 10.1016/j.catena.2023.107596.

[CR7] Bizzi S, Tangi M, Schmitt RJP, Pitlick J, Piégay H, Castelletti AF (2021) Sediment transport at the network scale and its link to channel morphology in the braided Vjosa River system. Earth Surf Process Land 46:2946–2962. 10.1002/esp.5225.

[CR8] Blott SJ, Pye K (2001) GRADISTAT: a grain size distribution and statistics package for the analysis of unconsolidated sediments. Earth Surf Process Land 26:1237–1248. 10.1002/esp.261.

[CR9] Borrelli P, Robinson DA, Fleischer LR, Lugato E, Ballabio C, Alewell C, Meusburger K, Modugno S, Schütt B, Ferro V, Bagarello V, van, Oost K, Montanarella L, Panagos P (2017) An assessment of the global impact of 21^st^ century land use change on soil erosion. Nat Commun 8:2013. 10.1038/s41467-017-02142-7. PMC5722879 29222506

[CR10] Brandt S (2000) Classification of geomorphological effects downstream of dams. CATENA 40:375–401. 10.1016/S0341-8162(00)00093-X.

[CR11] Campos JA, Pedrollo OC (2023) Modelling and assessing how small hydropower facilities affect sediment transport by using fuzzy inference systems. J Hydrol 620:129374. 10.1016/j.jhydrol.2023.129374.

[CR12] Casserly CM, Turner JN, O’Sullivan JJ, Bruen M, Bullock C, Atkinson S, Kelly-Quinn M (2020) Impact of low-head dams on bedload transport rates in coarse-bedded streams. Sci Total Environ 716: 136908. 10.1016/j.scitotenv.2020.136908. 32069694

[CR13] Casserly CM, Turner JN, O’ Sullivan JJ, Bruen M, Bullock C, Atkinson S, Kelly-Quinn M (2021) Effect of low-head dams on reach-scale suspended sediment dynamics in coarse-bedded streams. J Environ Manag 277:111452. 10.1016/j.jenvman.2020.111452. 33075653

[CR14] CEPF (Critical Ecosystem Partnership Fund) (2017) Ecosystem Profile: Mountains of Central Asia Biodiversity Hotspot. http://aarhus.kg/wp-content/uploads/2017/11/2_eng.pdf.

[CR15] Church M, Ferguson RI (2015) Morphodynamics: Rivers beyond steady state. Water Resour Res 51:1883–1897. 10.1002/2014WR016862.

[CR16] Cohen S, Kettner AJ, Syvitski JP, Fekete BM (2013) WBMsed, a distributed global-scale riverine sediment flux model: Model description and validation. Comput Geosci 53:80–93. 10.1016/j.cageo.2011.08.011.

[CR17] Cohen S, Kettner AJ, Syvitski JP (2014) Global suspended sediment and water discharge dynamics between 1960 and 2010: Continental trends and intra-basin sensitivity. Glob Planet Change 115:44–58. 10.1016/j.gloplacha.2014.01.011.

[CR18] Cohen S, Syvitski J, Ashley T, Lammers R, Fekete B, Li H-Y (2022) Spatial Trends and Drivers of Bedload and Suspended Sediment Fluxes in Global Rivers. Water Resour Res 58 10.1029/2021WR031583.

[CR19] Csiki S, Rhoads BL (2010) Hydraulic and geomorphological effects of run-of-river dams. Prog Phys Geogr: Earth Environ 34:755–780. 10.1177/0309133310369435.

[CR20] De Keyser J, Hayes DS, Marti B, Siegfried T, Seliger C, Schwedhelm H, Anarbekov O, Gafurov Z, López Fernández RM, Ramos Diez I, Alapfy B, Carey J, Karimov B, Karimov E, Wagner B, Habersack H (2023) Integrating Open-Source Datasets to Analyze the Transboundary Water–Food–Energy–Climate Nexus in Central Asia. Water 15:3482. 10.3390/w15193482.

[CR21] De Keyser J, Seliger C, Hayes DS, Schwedhelm H, Osuna Fuentes P, Carey JI, Siegfried T, Marti B, Habersack H (2026a) A regional-scale framework for assessing sustainable hydropower potential based on open-access data: the case of Central Asia. Appl Energy 414: 127843. 10.1016/j.apenergy.2026.127843.

[CR22] De Keyser J, Osuna Fuentes P, Hayes DS, Habersack H (2026b) A review of hydropower in Central Asia: Past, present, and future. Renew Sustain Energy Rev 226:116239. 10.1016/j.rser.2025.116239.

[CR23] De Keyser J, Ries W, Seliger C, Carey J, Hayes D, Arreaga Espin JV, Habersack H (2026c) HydroPlan-CA — A Hydropower Decision Support System for Central Asia. 10.5281/zenodo.21037792.

[CR24] Efthymiou NP, Palt S, Annandale GW, Karki P (2003) RESCON - Reservoir Conservation Model: Economic and Engineering Evaluation of Alternative Sediment Management Strategies. World Bank, Washington, DC. https://www.hydropower.org/sediment-management-resources/tool-reservoir-conservation-model-rescon-2-beta.

[CR25] EI (Environmental Intelligence) Team, Politecnico di Milano (2021) CASCADE: A toolbox for network-scale connectivity assessment. [Software], available at: http://www.cascademodel.org/.

[CR26] Fantin-Cruz I, de Oliveira MD, Campos JA, de Campos MM, Souza Ribeiro L, de Mingoti R, de Souza ML, Pedrollo O, Hamilton SK (2020) Further Development of Small Hydropower Facilities Will Significantly Reduce Sediment Transport to the Pantanal Wetland of Brazil. Front Environ Sci 8 10.3389/fenvs.2020.577748.

[CR27] Fencl JS, Mather ME, Costigan KH, Daniels MD (2015) How Big of an Effect Do Small Dams Have? Using Geomorphological Footprints to Quantify Spatial Impact of Low-Head Dams and Identify Patterns of Across-Dam Variation. PLoS One 10:e0141210. 10.1371/journal.pone.0141210. PMC4634923 26540105

[CR28] Gabbud C, Lane SN (2016) Ecosystem impacts of Alpine water intakes for hydropower: the challenge of sediment management. WIREs Water 3:41–61. 10.1002/wat2.1124.

[CR29] Gorelick N, Hancher M, Dixon M, Ilyushchenko S, Thau D, Moore R (2017) Google Earth Engine: Planetary-scale geospatial analysis for everyone. Remote Sens Environ 202:18–27. 10.1016/j.rse.2017.06.031.

[CR30] Hansen WF (2001) Identifying stream types and management implications. For Ecol Manag 143:39–46. 10.1016/S0378-1127(00)00503-X.

[CR31] Hatono M, Yoshimura K (2020) Development of a global sediment dynamics model. Prog Earth Planet Sci 7 10.1186/s40645-020-00368-6.

[CR32] Hauer C, Leitner P, Unfer G, Pulg U, Habersack H, Graf W (2018) The Role of Sediment and Sediment Dynamics in the Aquatic Environment. In: Schmutz S, Sendzimir J (eds) Riverine Ecosystem Management. Springer International Publishing, Cham, pp 151–169. 10.1007/978-3-319-73250-3_8.

[CR33] Hayes DS, Hägele T, Kopecki I, Zeiringer B, Karimov E, Karimov BK, Coeck J, Verhelst P, De Keyser J, Omonov O, Schneider M (2025) Environmental flows assessment integrating snow trout habitat requirements in the Shakhimardan basin, Central Asia. Int J Environ Sci Technol. 10.1007/s13762-025-06761-2.

[CR34] Inman DL (1952) Measures for Describing the Size Distribution of Sediments. J Sediment Res 22. 10.1306/D42694DB-2B26-11D7-8648000102C1865D.

[CR35] ISO: DIN EN ISO 14688-1:2017-02 (2017) Geotechnical investigation and testing: Identification and classification of soil. Deutsches Institut für Normung e.V

[CR36] Karthe D, Chalov S, Borchardt D (2015) Water resources and their management in central Asia in the early twenty first century: status, challenges and future prospects. Environ Earth Sci 73:487–499. 10.1007/s12665-014-3789-1.

[CR37] Kondolf GM (1997) PROFILE: Hungry Water: Effects of Dams and Gravel Mining on River Channels. Environ Manag 21:533–551. 10.1007/s002679900048. 9175542

[CR38] Langhorst T, Pavelsky T (2023) Global Observations of Riverbank Erosion and Accretion From Landsat Imagery. J Geophys Res Earth Surf 128. 10.1029/2022JF006774.

[CR39] Lehner B, Verdin K, Jarvis A (2008) New Global Hydrography Derived From Spaceborne Elevation Data. EoS Trans 89:93–94. 10.1029/2008eo100001.

[CR40] Leopold LB (1995) A view of the river, 3rd edn. Harvard Univ. Press, Cambridge, Mass

[CR65] Li D, Lu X, Overeem I, Walling DE, Syvitski J, Kettner AJ, Bookhagen B, Zhou Y, Zhang T (2021) Exceptional increases in fluvial sediment fluxes in a warmer and wetter High Mountain Asia. Science 374:599–603. 10.1126/science.abi9649. 34709922

[CR41] Li H-Y, Tan Z, Ma H, Zhu Z, Abeshu GW, Zhu S, Cohen S, Zhou T, Xu D, Leung LR (2022a) A new large-scale suspended sediment model and its application over the United States. Hydrol Earth Syst Sci 26:665–688. 10.5194/hess-26-665-2022.

[CR42] Li D, Lu X, Walling DE, Zhang T, Steiner JF, Wasson RJ, Harrison S, Nepal S, Nie Y, Immerzeel WW, Shugar DH, Koppes M, Lane S, Zeng Z, Sun X, Yegorov A, Bolch T (2022b) High Mountain Asia hydropower systems threatened by climate-driven landscape instability. Nat Geosci 15:520–530. 10.1038/s41561-022-00953-y.

[CR43] Magilligan FJ, Roberts MO, Marti M, Renshaw CE (2021) The impact of run-of-river dams on sediment longitudinal connectivity and downstream channel equilibrium. Geomorphology 376: 107568. 10.1016/j.geomorph.2020.107568.

[CR44] Marti B, Siegfried T, Yakovlev A, Zhumabaev A, Karger D, Wakil AW, Ragettli S (2023a) CA-discharge data set, scripts and raw data. 10.5281/zenodo.8147591. PMC10477246 37666883

[CR45] Marti B, Yakovlev A, Karger DN, Ragettli S, Zhumabaev A, Wakil AW, Siegfried T (2023b) CA-discharge: Geo-Located Discharge Time Series for Mountainous Rivers in Central Asia. Sci Data 10: 579. 10.1038/s41597-023-02474-8. PMC10477246 37666883

[CR46] Moragoda N, Cohen S (2020) Climate-induced trends in global riverine water discharge and suspended sediment dynamics in the 21^st^ century. Glob Planet Change 191:103199. 10.1016/j.gloplacha.2020.103199.

[CR47] Mulligan M, van Soesbergen A, Sáenz L (2020) GOODD, a global dataset of more than 38,000 georeferenced dams. Sci Data 7: 31. 10.1038/s41597-020-0362-5. PMC6972789 31964896

[CR48] Nilsson C, Reidy CA, Dynesius M, Revenga C (2005) Fragmentation and flow regulation of the world’s large river systems. Science 308:405–408. 10.1126/science.1107887. 15831757

[CR49] Ouellet Dallaire C, Lehner B, Sayre R, Thieme M (2019) A multidisciplinary framework to derive global river reach classifications at high spatial resolution. Environ Res Lett 14:24003. 10.1088/1748-9326/aad8e9.

[CR50] Parker G, Klingeman PC (1982) On why gravel bed streams are paved. Water Resour Res 18:1409–1423. 10.1029/WR018i005p01409.

[CR51] Pearson AJ, Pizzuto J (2015) Bedload transport over run-of-river dams, Delaware, U.S.A. Geomorphology 248:382–395. 10.1016/j.geomorph.2015.07.025.

[CR52] Schmitt RJP, Bizzi S, Castelletti A (2016) Tracking multiple sediment cascades at the river network scale identifies controls and emerging patterns of sediment connectivity. Water Resour Res 52:3941–3965. 10.1002/2015WR018097.

[CR53] Schmitt RJP, Bizzi S, Castelletti A, Kondolf GM (2018a) Improved trade-offs of hydropower and sand connectivity by strategic dam planning in the Mekong. Nat Sustain 1:96–104. 10.1038/s41893-018-0022-3.

[CR54] Schmitt RJP, Bizzi S, Castelletti AF, Kondolf GM (2018b) Stochastic Modeling of Sediment Connectivity for Reconstructing Sand Fluxes and Origins in the Unmonitored Se Kong, Se San, and Sre Pok Tributaries of the Mekong River. J Geophys Res Earth Surf 123:2–25. 10.1002/2016JF004105.

[CR55] Schmitt RJP, Bizzi S, Castelletti A, Opperman JJ, Kondolf GM (2019) Planning dam portfolios for low sediment trapping shows limits for sustainable hydropower in the Mekong. Sci Adv 5: eaaw2175. 10.1126/sciadv.aaw2175. PMC6984971 32047852

[CR56] Schmutz S, Moog O (2018) Dams: Ecological Impacts and Management. In: Schmutz S, Sendzimir J (eds) Riverine Ecosystem Management. Springer International Publishing, Cham, pp 111–127. 10.1007/978-3-319-73250-3_6.

[CR57] Tangi M, Schmitt R, Bizzi S, Castelletti A (2019) The CASCADE toolbox for analyzing river sediment connectivity and management. Environ Model Softw 119:400–406. 10.1016/j.envsoft.2019.07.008.

[CR58] Uzhydromet (2021) Weighted sediment discharge, Table 1.7 (Расходы взвешенных наносов, Таблица 1.7.). Hydrometeorological Service of the Republic of Uzbekistan

[CR59] Whittemore A, Ross MRV, Dolan W, Langhorst T, Yang X, Pawar S, Jorissen M, Lawton E, Januchowski-Hartley S, Pavelsky T (2020) A Participatory Science Approach to Expanding Instream Infrastructure Inventories. Earth’s Future 8. 10.1029/2020EF001558.

[CR60] Winemiller KO, McIntyre PB, Castello L, Fluet-Chouinard E, Giarrizzo T, Nam S, Baird IG, Darwall W, Lujan NK, Harrison I, Stiassny MLJ, Silvano RAM, Fitzgerald DB, Pelicice FM, Agostinho AA, Gomes LC, Albert JS, Baran E, Petrere M, Zarfl C, Mulligan M, Sullivan JP, Arantes CC, Sousa LM, Koning AA, Hoeinghaus DJ, Sabaj M, Lundberg JG, Armbruster J, Thieme ML, Petry P, Zuanon J, Torrente Vilara G, Snoeks J, Ou C, Rainboth W, Pavanelli CS, Akama A, van Soesbergen A, Sáenz L (2016) Balancing hydropower and biodiversity in the Amazon, Congo, and Mekong. Science 351:128–129. 10.1126/science.aac7082. 26744397

[CR61] Yamazaki D, Ikeshima D, Sosa J, Bates PD, Allen GH, Pavelsky TM (2019) MERIT Hydro: A High-Resolution Global Hydrography Map Based on Latest Topography Dataset. Water Resour Res 55:5053–5073. 10.1029/2019WR024873.

[CR62] Yang X, Pavelsky TM, Allen GH, Donchyts G (2020) RivWidthCloud: An Automated Google Earth Engine Algorithm for River Width Extraction From Remotely Sensed Imagery. IEEE Geosci Remote Sens Lett 17:217–221. 10.1109/LGRS.2019.2920225.

[CR63] Yang X, Pavelsky TM, Ross MRV, Januchowski-Hartley SR, Dolan W, Altenau EH, Belanger M, Byron D, Durand M, van Dusen I, Galit H, Jorissen M, Langhorst T, Lawton E, Lynch R, Mcquillan KA, Pawar S, Whittemore A (2022) Mapping Flow-Obstructing Structures on Global Rivers. Water Resour Res 58. 10.1029/2021WR030386.

[CR64] Zonn IS, Zhiltsov SS, Kostianoy AG, Semenov AV (eds) (2020) Water Resources Management in Central Asia. In: The Handbook of Environmental Chemistry. Springer International Publishing, Cham. 10.1007/978-3-030-57986-9.

