# Supplementary material for: Modeling Sediment Transport Potential and Connectivity to Support Sustainable Hydropower Development in Central Asia
Source: Environ Manage. 2026 Sep 15;76(9):313. doi: 10.1007/s00267-026-02598-8 (PMC13577999; doi:10.1007/s00267-026-02598-8)
Supplement: Supplementary file 1 [file 267_2026_2598_MOESM1_ESM.docx]

**Supplementary Material**

Supplement 1: Channel-Forming Discharge

Sediment transport, especially bedload, is primarily linked to high-flow conditions when the hydraulic conditions result in shear forces exceeding a certain critical value. To investigate bedload transport, we needed to select a corresponding discharge value for which sediment transport is expected to occur. Literature refers to this discharge value as “effective” or “dominant” discharge and is often linked to the bankfull discharge (Church and Ferguson 2015). Leopold (1995) associates the bankfull discharge required for sediment transport with high-flow events that have a return period of 1 to 2.5 years. With the assumption that sediment transport influences channel form annually during high-flow events, we developed a correlation as follows to extract discharge percentiles from the Flo1K dataset (Barbarossa et al. 2018) for each river reach. These discharge percentiles are calculated by multiplying a correction factor by the mean monthly value from the Flo1K dataset (Barbarossa et al. 2018). The correction factor is a function of the seasonal variation index (SVI), which is obtained by correlating the percentiles of the time series from the CA-discharge dataset to the seasonal variation index. We selected Q5 (discharge with a probability of exceedance of 5% of the used mean monthly values). Figure 1 shows the correlation between the correction factor for Q5 and the SVI for the gauging stations, for which time series data are included in the CA-discharge dataset (Marti et al. 2023a). An overall R² of 0.9 was calculated, supporting the use of this correction factor to calculate the Q5 values for all river reaches in the study area.

Fig. 1 Function developed for the correction factor for the Q5 value (5% exceedance probability) calculated from the seasonal variability index (SVI) using data from the CA-discharge dataset (Marti et al. 2023a)

Supplement 2: Estimating Month of Peak Discharge

We obtained river width information from Sentinel data using a modified version of the RivWidthCloud algorithm (Yang et al. 2020). It was necessary to select river width information when the discharge is highest during the year to correspond to the channel-forming discharge values. Therefore, we extracted the month of peak discharge for the gauged data of the CA-discharge dataset (Marti et al. 2023a) by calculating the maximum discharge per year, following the filtering methodology described in their study to calculate mean discharge values. We then scored the month of the expected maximum discharge following the methodology described in Altenau et al. (2021). Using the elevation information (Jarvis et al. 2008a) of the catchment represented by each gauging station, we identified a correlation between the month of the peak discharge and the mean elevation of the catchments of the Central Asian rivers (Figure 2). After filtering the gauging stations as described in Marti et al. (2023b), we obtained 122 gauging stations, of which only five indicated March, August, September or November, which we neglected in the following steps. We observed that for catchments with a mean elevation below 2000 m, April was identified as the month of peak discharge, whereas for elevations between 2000 and 2500 m, May and April are the months of peak discharge. Also, peak discharges were observed for elevations between 2500 and 3000 m in June and above 3000 m in July. These observations are supported by the seasonal development of discharge and the associated runoff regime classification observed in the CA-discharge dataset (Marti et al. 2023b).

Fig. 2 Correlation between the month of the peak discharge and the mean elevation of the catchments of the Central Asian rivers using data of gauging stations included in Marti et al. (2023a) and elevation information from digital elevation models (Jarvis et al. 2008a)

Applying these relations between the elevation and the month of the maximum discharge, we used the extents of sub-catchments (level 7, Lehner et al. 2008) to extract their mean elevation (Jarvis et al. 2008b) and merged this information with the extents of the Sentinel-2 tiles (extents from Meyers 2015). For each Sentinel-2 tile covering our four river basins, we hence obtained an associated month for which the river width is expected to be at its maximum. Using this information, we extract river width information from the modified RivWidthCloud algorithm.

Supplement 3: Example Results Shakimardan and Suyok

To illustrate the application of our geospatial dataset, we extracted the data for two sites here: Shakimardan and Suyok (compare main text Table 2).

Table 1: Extracted values from the geospatial dataset for the sites Shakimardan and Suyok

| Shakimardan | | | | | | | |
| --- | --- | --- | --- | --- | --- | --- | --- |
| Reach ID | Transport Probability [%] | | Entrainment [%] | | Deposition [%] | | Connectivity [-] |
| 507089 | Silt | 91.3 | Silt | 0.0 | Silt | 0.0 | 0.92 |
|  | Sand | 93.8 | Sand | 0.0 | Sand | 0.0 |  |
|  | Gravel | 71.6 | Gravel | 0.0 | Gravel | 0.0 |  |
|  | Cobbles | 46.9 | Cobbles | 0.0 | Cobbles | 0.0 |  |
|  | Boulders | 8.5 | Boulders | 0.0 | Boulders | 0.0 |  |
| 507088 | Silt | 91.3 | Silt | 0.0 | Silt | 90.6 | 0.92 |
|  | Sand | 93.8 | Sand | 0.0 | Sand | 89.4 |  |
|  | Gravel | 71.7 | Gravel | 0.0 | Gravel | 68.8 |  |
|  | Cobbles | 46.9 | Cobbles | 0.0 | Cobbles | 40.5 |  |
|  | Boulders | 8.9 | Boulders | 0.0 | Boulders | 0.0 |  |
| 507087 | Silt | 91.3 | Silt | 0.0 | Silt | 91.3 | 0.93 |
|  | Sand | 93.6 | Sand | 13.1 | Sand | 79.6 |  |
|  | Gravel | 70.3 | Gravel | 31.0 | Gravel | 41.3 |  |
|  | Cobbles | 43.7 | Cobbles | 46.0 | Cobbles | 0.0 |  |
|  | Boulders | 5.0 | Boulders | 6.8 | Boulders | 0.0 |  |
|  | | | | | | | |
| Suyok | | | | | | | |
| Reach ID | Transport Probability [%] | | Entrainment [%] | | Deposition [%] | | Connectivity [-] |
| 431249 | Silt | 91.3 | Silt | 89.2 | Silt | 0.0 | 0.57 |
|  | Sand | 94.1 | Sand | 76.9 | Sand | 16.4 |  |
|  | Gravel | 82.1 | Gravel | 36.7 | Gravel | 45.8 |  |
|  | Cobbles | 68.2 | Cobbles | 0.0 | Cobbles | 67.2 |  |
|  | Boulders | 49.7 | Boulders | 0.0 | Boulders | 49.3 |  |

The Shakimardan site covers three reaches of our dataset, whereas the Suyok site consists of only one.

In general, transport probabilities are higher at the Suyok site, with medium to very high probabilities across all sediment sizes. At the Shakimardan site, transport probabilities are very low for boulders, low cobbles, high for gravel, and very high for sand and silt.

At the Shakimardan site, entrainment and deposition patterns vary between reaches. The upper reach (507088) shows no potential for entrainment or deposition. Entrainment is only expected in the lower reach, with very low probability for boulders and sand, low for gravel, and medium for cobbles. In the middle reach (507088), deposition probabilities are very high for silt and sand, high for gravel, and medium for cobbles. The lower reach (507087) exhibits very high deposition probabilities for silt, high for sand, and moderate for gravel.

At the Suyok site, entrainment may occur with low probabilities for gravel and high to very high probabilities for sand and silt, respectively. Deposition, on the other hand, is expected for boulders (medium), cobbles (high), gravel (medium), and sand and silt (low) probabilities.

The connectivity index is very high for the Shakimardan site across all reaches (above 0.92) and lower for Suyok site (0.57).

References

Altenau EH, Pavelsky TM, Durand MT, Yang X, Frasson RPdM, Bendezu L (2021) The Surface Water and Ocean Topography (SWOT) Mission River Database (SWORD): A Global River Network for Satellite Data Products. Water Resour. Res. 57. https://doi.org/10.1029/2021WR030054

Barbarossa V, Huijbregts MAJ, Beusen AHW, Beck HE, King H, Schipper AM (2018) FLO1K, global maps of mean, maximum and minimum annual streamflow at 1 km resolution from 1960 through 2015. Sci Data 5:180052. https://doi.org/10.1038/sdata.2018.52

Church M, Ferguson RI (2015) Morphodynamics: Rivers beyond steady state. Water Resour. Res. 51:1883–1897. https://doi.org/10.1002/2014WR016862

Jarvis A, Reuter HI, Nelson A, Guevara E (2008a) Hole-filled seamless SRTM data V4, International Centre for Tropical Agriculture (CIAT). https://srtm.csi.cgiar.org

Jarvis A, Reuter HI, Nelson A, Guevara E (2008b) Hole-filled seamless SRTM data V4., International Centre for Tropical Agriculture (CIAT). https://srtm.csi.cgiar.org. Accessed 29 July 2024. Accessed 29 July 2024

Lehner B, Verdin K, Jarvis A (2008) New Global Hydrography Derived From Spaceborne Elevation Data. EoS Transactions 89:93–94. https://doi.org/10.1029/2008eo100001

Leopold LB (1995) A view of the river, 3^rd^ edn. Harvard Univ. Press, Cambridge, Mass.

Marti B, Siegfried T, Yakovlev A, Zhumabaev A, Karger D, Wakil AW, Ragettli S (2023a) CA-discharge data set, scripts and raw data. https://doi.org/10.5281/zenodo.8147591

Marti B, Yakovlev A, Karger DN, Ragettli S, Zhumabaev A, Wakil AW, Siegfried T (2023b) CA-discharge: Geo-Located Discharge Time Series for Mountainous Rivers in Central Asia. Sci Data 10:579. https://doi.org/10.1038/s41597-023-02474-8

Meyers JE (2015) Sentinel-2-Shapefile-Index: GitHub. https://github.com/justinelliotmeyers/Sentinel-2-Shapefile-Index. Accessed 5 August 2025

Yang X, Pavelsky TM, Allen GH, Donchyts G (2020) RivWidthCloud: An Automated Google Earth Engine Algorithm for River Width Extraction From Remotely Sensed Imagery. IEEE Geosci. Remote Sensing Lett. 17:217–221. https://doi.org/10.1109/LGRS.2019.2920225
